# Tell the Difference Between Mitosis and Meiosis: Interplay Between Chromosomes, Cytoskeleton, and Cell Cycle Regulation

**DOI:** 10.3389/fcell.2021.660322

**Published:** 2021-04-08

**Authors:** Masamitsu Sato, Yasutaka Kakui, Mika Toya

**Affiliations:** ^1^Laboratory of Cytoskeletal Logistics, Center for Advanced Biomedical Sciences (TWIns), Waseda University, Tokyo, Japan; ^2^Institute for Advanced Research of Biosystem Dynamics, Waseda Research Institute for Science and Engineering, Graduate School of Advanced Science and Engineering, Waseda University, Tokyo, Japan; ^3^Institute for Medical-Oriented Structural Biology, Waseda University, Tokyo, Japan; ^4^Waseda Institute for Advanced Study, Waseda University, Tokyo, Japan; ^5^Major in Bioscience, Global Center for Science and Engineering, Faculty of Science and Engineering, Waseda University, Tokyo, Japan

**Keywords:** mitosis, meiosis, microtubule, kinetochore, cell cycle, fission yeast (*Schizosaccharomyces pombe*), chromosome

## Abstract

Meiosis is a specialized style of cell division conserved in eukaryotes, particularly designed for the production of gametes. A huge number of studies to date have demonstrated how chromosomes behave and how meiotic events are controlled. Yeast substantially contributed to the understanding of the molecular mechanisms of meiosis in the past decades. Recently, evidence began to accumulate to draw a perspective landscape showing that chromosomes and microtubules are mutually influenced: microtubules regulate chromosomes, whereas chromosomes also regulate microtubule behaviors. Here we focus on lessons from recent advancement in genetical and cytological studies of the fission yeast *Schizosaccharomyces pombe*, revealing how chromosomes, cytoskeleton, and cell cycle progression are organized and particularly how these are differentiated in mitosis and meiosis. These studies illuminate that meiosis is strategically designed to fulfill two missions: faithful segregation of genetic materials and production of genetic diversity in descendants through elaboration by meiosis-specific factors in collaboration with general factors.

## Introduction

Eukaryotic cells undergo two styles of cell division. Mitosis is a type of cell division for somatic cells and for the asexual reproduction of unicellular eukaryotic cells. Meiosis is the type of cell division for the production of gametes in sexual reproduction. How mitosis and meiosis are differentially designed and conducted is a long-standing key question in the field of cell biology. Yeast cells undergo both types of divisions that can be switched according to environmental conditions, and therefore yeast cells have been studied to reveal underlying molecular mechanisms. In the last few decades, a considerable number of studies revealed that cells exploit meiosis-specific factors to shift the division style from the standard mitotic one to the specialized meiotic one. We, in this review, focus on how such meiosis-specific factors dramatically modulate the way of divisions, featuring fission yeast as an example.

## Overview of Chromosome Configuration in Mitosis and Meiosis

**FIGURE 1:**
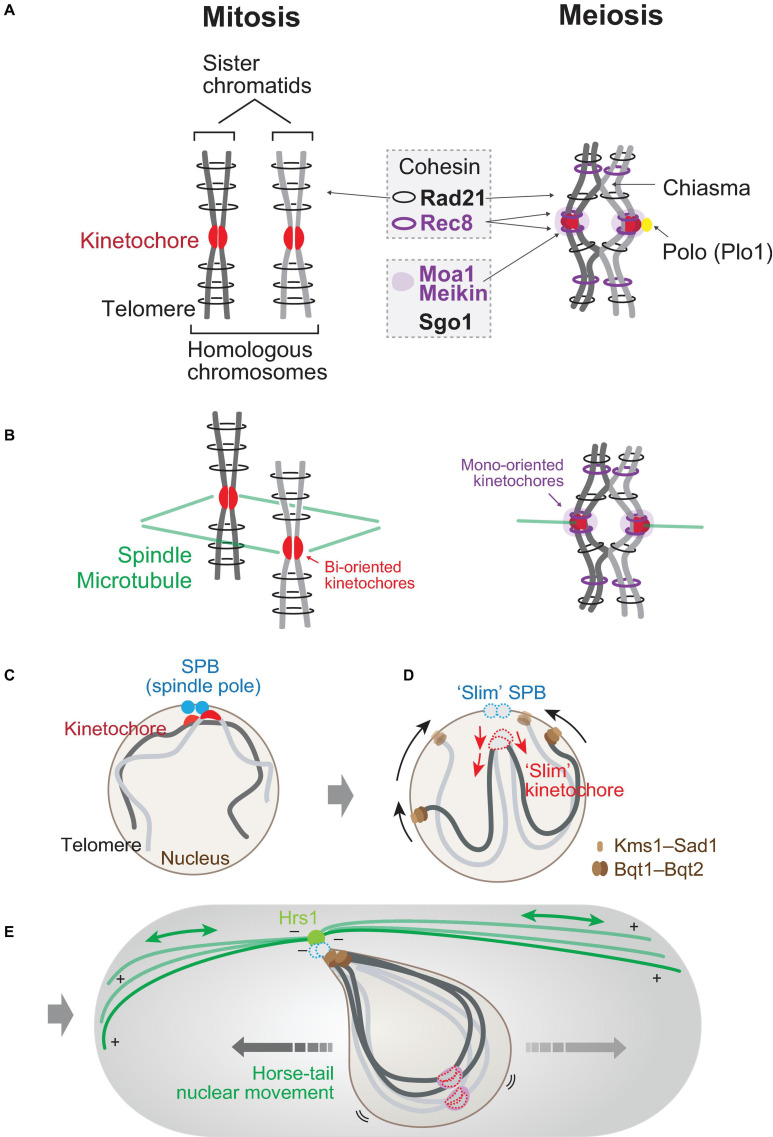
Chromosomes in mitosis and in meiosis. **(A)** Chromosomes in mitosis and in meiosis. During mitotic cell cycles (left), homologous chromosomes behave independently. Sister chromatids are connected by mitotic cohesin Rad21 (Scc1). In meiosis I (right), homologous chromosomes are paired and crossed *via* chiasmata. Rad21 locates in the arm region of chromosomes in both divisions, whereas meiotic cohesin Rec8 (purple ring) locates in both the arm and centromeric regions. Moa1 (Meikin) is a meiosis-specific kinetochore protein that protects centromeric Rec8 cohesin through the recruitment of shugoshin (Sgo1) to centromeres and regulates attachment to microtubules through the recruitment of Polo kinase (Plo1). **(B)** Orientation of kinetochores. In mitosis (left), sister kinetochores are bi-oriented in a back-to-back position. In meiosis I, sister kinetochores are oriented in a side-by-side position. Spindle microtubules, green. **(C–E)** Conversion of chromosome arrangement upon sexual differentiation in fission yeast. In interphase (e.g., G1 phase) of mitotic cycles **(C)**, centromeres (kinetochores; red) are clustered at spindle pole bodies (SPBs; blue). In reaction to mating pheromone **(D)**, telomeres get clustered to SPBs. Telomeres slide on the nuclear envelope toward SPBs *via* interaction with meiosis-specific bouquet proteins (Bqt1 and Bqt2) and nuclear membrane proteins (Kms1/2–Sad1). Kinetochores are dissociated off SPBs. A number of kinetochore and SPB components are dissociated in this stage (slim SPBs and kinetochores). During meiotic prophase **(E)**, a cytoplasmic array of microtubules is tethered by Hrs1 at SPBs, thereby shaking the nucleus. The SPB-led back-and-forth movement of the nucleus is called horse-tail nuclear movement.

As one of the most evident and essential differences in mitosis and meiosis is configuration of chromosomes, we briefly give an overview on how chromosomes differ in those divisions. In both divisions, chromosomes are duplicated in the S phase of the cell cycle, resulting in forming a pair of the replicated chromosomes defined as sister chromatids (Figure 1A). During DNA replication, cohesion between sister chromatids is established by the cohesin complex, which forms a proteinaceous ring comprised of two coiled-coil components, Psm1^*SMC*1^ and Psm3^*SMC*3^, kleisin/Rad21, and HEAT repeat Psc3^*SCC*3^.

The molecular basis constituting meiosis-specific chromosome configuration is largely attributed to meiotic cohesin, in which kleisin subunit Rad21 is replaced by Rec8 (Parisi et al., 1999; Watanabe and Nurse, 1999). Rec8 is a cohesin expressed specifically during meiosis, and both cohesins are conserved in all eukaryotes from yeast to human [reviewed in Ishiguro (2019)]. In meiosis, meiotic Rec8-cohesin, in addition to mitotic Rad21-cohesin, decorates chromosomes to connect sister chromatids as well as homologous chromosomes which are linked by chiasmata. The difference of Rec8-cohesin and Rad21-cohesin is their chromosomal localization. Rec8-cohesin can localize at the core centromeric region but Rad21-cohesin cannot in both mitosis and meiosis. Furthermore, Rec8-cohesin forms two distinct complexes, one with Psc3 for centromeric cohesion and the other with arm cohesion (Kitajima et al., 2003).

The kinetochore is assembled at the centromere of a chromosome, which serves as a dock site for spindle microtubule emanated from the spindle pole (Figure 1B). In mitosis, homologous chromosomes (a pair of maternal and paternal chromosomes) in diploid cells behave independently upon segregation, whereas in meiosis, homologous chromosomes are paired and a part of them are physically connected by chiasmata.

In mitosis, sister kinetochores built on core centromeres of sister chromatids are oppositely oriented in a back-to-back manner (bi-oriented; Figure 1B). On the other hand, the orientation in meiosis is converted to mono-orientation, in which the sister kinetochores are united in a side-by-side manner (Figure 1B). The mono-orientation is constructed by Rec8-cohesin and monopolin proteins (Moa1 in fission yeast; Spo13 and Mam1 in budding yeast) (Lee et al., 2002; Shonn et al., 2002; Katis et al., 2004; Yokobayashi and Watanabe, 2005; Monje-Casas et al., 2007; Kim et al., 2015; Galander et al., 2019).

Moa1 is a meiosis-specific kinetochore protein and is later found to be a member of the Meikin family together with Spo13 in budding yeast (Kim et al., 2015). Moa1 together with the Rec8-cohesin located at core centromeres ties up kinetochores of sister chromatids facing toward the same direction so that the sister kinetochores can be mono-oriented (meiosis, Figure 1B; Yokobayashi and Watanabe, 2005; Kim et al., 2015). The mono-orientation of sister kinetochores is maintained until anaphase of meiosis I (anaphase I) to ensure reductional division but is resolved to the mitotic bi-oriented style by the onset of meiosis II (equational division).

In mutants lacking Rec8 or Moa1, sister kinetochores are split and bi-oriented, and the division pattern of meiosis I results in equational division, as is seen in wild-type mitosis and meiosis II. Thus, mono-orientation of kinetochores mediated by Rec8 and Moa1 is essential for the establishment of reductional division in meiosis I (Figure 1B).

In the absence of Moa1 (or Meikin), localization of the Shugoshin protein (Sgo1 in fission yeast meiosis and SGO2 or SGOL2 in mouse meiosis) diminished (Kim et al., 2015). As shugoshin protein protects the cleavage of meiotic cohesin Rec8 during meiosis I, *moa1*Δ (the *moa1* deletion mutant) cells lose Rec8 at centromeres as a result. Thus, meiotic kinetochore protein Meikin constitutes the mono-orientation of sister kinetochores as well as protects meiotic cohesin at centromeres through the recruitment of shugoshin. Shugoshin recruits PP2A to counteract the phosphorylation of the kleisin subunit of the cohesin complex to prevent cleavage (Kitajima et al., 2006; Riedel et al., 2006).

These two functions of Meikin are conducted *via* Polo kinase. Polo kinase is one of the mitotic kinases which mainly localize to spindle poles [reviewed in Nigg (2001) and Zitouni et al. (2014)]. Plo1, the *Schizosaccharomyces pombe* Polo kinase, also localizes to SPBs during mitosis but localizes to meiotic kinetochores using Moa1 as a platform (Figure 1A; Kakui et al., 2013; Kim et al., 2015). Polo kinase at meiotic kinetochores with Moa1 thus dictates the mono-orientation of sister kinetochores *via* Rec8 (or monopolin in budding yeast) as well as the protection of centromeric cohesin *via* shugoshin. In addition to those dual functions of Moa1–Plo1, we will later discuss additional roles regarding interaction with microtubules.

## Alteration of Chromosome Arrangement in the Nucleus Upon Sexual Differentiation

A number of studies revealed that the geographical arrangement of chromosomes in the nucleus affects the behaviors of chromosomes essential to accomplish meiotic events. Most importantly, the location of the chromosomes in the nucleus directly affects the efficiency of meiotic recombination. A brief schematic overview of chromosome allocation in the nucleus of the fission yeast is summarized in Figures 1C–E.

In the interphase of mitotic cell cycles, the centromeres of all chromosomes are clustered near SPBs (the centrosome equivalent in yeast species) located at the nuclear periphery (Rabl orientation, Figure 1C; Funabiki et al., 1993). *S. pombe* cells undergo sexual differentiation when cells are starved under nitrogen depletion and when the ploidy of cells is in a diploid state originated from a pair of haploid cells with two distinct mating types (Yamamoto et al., 1997). Zygotic meiosis occurs when a pair of haploid cells is fused through the mating process to form a diploid cell right before entry into meiosis. In contrast, azygotic meiosis occurs when proliferating cells in a diploid state start meiosis without the mating process (Cipak et al., 2014).

Both in zygotic and azygotic meiosis the mating pheromones are secreted and received on the cell surface to induce differentiation *via* the MAP kinase cascade.

The mating pheromone–MAPK pathway affects chromosome positioning at the initial stage of sexual differentiation. First, as illustrated in Figure 1D, telomeres are clustered in reaction to the mating pheromone. Then, centromeres are dissociated from the SPBs after cell conjugation in the case of zygotic meiosis (Chikashige et al., 1997; Jin et al., 1998). This means that the chromosome arrangement in the nucleus becomes upside-down from the original state (Rabl orientation): telomeres are clustered at SPBs, whereas centromeres (kinetochores) are located far from SPBs (Figure 1E). This state is called “bouquet” arrangement, and the upside-down allocation of chromosomes hung from SPBs is essential to promote pairing and meiotic recombination of homologous chromosomes in meiotic prophase. The bouquet configuration of chromosomes is highly conserved throughout eukaryotes, which is essential to promote meiotic recombination [reviewed in Scherthan (2001)].

A cytoplasmic array of microtubules is assembled particularly during meiotic prophase, and the minus ends of such microtubules are tethered at the SPBs. A meiosis-specific coiled-coil protein, Hrs1 (also known as Mcp6), localizes to SPBs and anchors the cytoplasmic array of microtubules at their minus ends (Figure 1E), which serves as a fulcrum at the SPBs that transmit the dynamic motion of microtubules to the oscillatory nuclear movement (Saito et al., 2005; Tanaka et al., 2005a; Funaya et al., 2012). The SPB-led microtubule array is dynamically reformed to pull and push the SPBs and the accompanying nucleus in the cytoplasm, by which the nucleus repeats a back-and-forth movement in the cytoplasm during meiotic prophase (Chikashige et al., 1994; Ding et al., 1998; Yamamoto et al., 1999; Hiraoka et al., 2000). The microtubule-driven “horse-tail nuclear movement” aligns the upside-down chromosome bouquet and is thus essential for promotion of pairing and recombination (Yamamoto et al., 1999; Niwa et al., 2000).

The molecular mechanisms for clustering of telomeres have been intensively studied, and meiosis-specific telomere proteins Bqt1 and Bqt2 (bouquet proteins) play central roles for telomere clustering. Bqt1–Bqt2 binds to the constitutive telomere protein Rap1 and also associates nuclear membrane proteins Sad1–Kms1 (and Kms2) (Figure 1D; Chikashige et al., 2006). Sad1 is an inner nuclear membrane protein containing the SUN domain, whereas Kms1/2 are outer ones with the KASH domain, and both domains are widely conserved among eukaryotes (Hagan and Yanagida, 1995; Shimanuki et al., 1997; Wälde and King, 2014).

The whole complex is called linker of nucleoskeleton and cytoskeleton (LINC), and it brings all the telomeres toward SPBs by sliding along the nuclear envelope using the cytoplasmic microtubules tethered at SPBs. The LINC complex in *S. pombe* includes the γ-tubulin complex, a base for microtubule nucleation, and the dynein motor protein complex [composed of heavy (Dhc1) and light (Dlc1) chains as well as dynactin (Ssm4)] (Yamashita et al., 1997; Yamamoto et al., 1999; Miki et al., 2002; Yoshida et al., 2013).

Systems to rearrange chromosome positions in eukaryotes are generally conserved: cytoskeleton such as actin (in the budding yeast *Saccharomyces cerevisiae*) or microtubule (in *S. pombe*, *Caenorhabditis elegans* and mice, and partly in *Drosophila melanogaster*) plays functions in the reorganization of chromosome states into the telomere-led bouquet arrangement (reviewed in Rubin et al., 2020). The function of the LINC complex (SUN-KASH proteins) is also conserved in eukaryotes. For instance, in mice germ cells, Majin serves as a related function in the linkage of telomeres and the nuclear envelope as a functional homolog of Bqt4, another transmembrane bouquet protein connecting telomeres and inner nuclear membrane (Chikashige et al., 2009). The transmembrane protein Majin interacts with SUN1-KASH5 proteins as well as with telomere-binding proteins TERB2-TERB1, thereby enhancing the association of telomeres to the nuclear envelope upon meiotic entry (Shibuya et al., 2015). Thus, SUN-KASH proteins are widely employed among eukaryotes to dynamically alter chromosome arrangement inside the nucleus during meiosis [reviewed in Chikashige et al. (2007) and Rubin et al. (2020)].

In addition, the telomere bouquet may regulate spindle functions. The first report described that if the bouquet formation is defective (e.g., in *bqt1*Δ or in *taz1*Δ; Taz1 is a telomere-binding protein), SPBs are fragmented, which results in defective spindles such as monopolar and multipolar ones (Tomita and Cooper, 2007). This is mostly due to the SPB-led horse-tail nuclear movement: SPBs are often apart from the main nuclear body after frequent shaking by cytoplasmic microtubules because the spindle phenotype can be rescued by interrupting the nuclear movement (Chikashige et al., 2014). In mitosis and meiosis, the association of centromeres to SPBs promote mitotic spindle formation (Fennell et al., 2015; Fernández-Álvarez et al., 2016). Taking this knowledge together, we can generalize that chromosome configuration controls the spindle.

Another study reported that bouquet formation contributes to the correct attachment of kinetochores and microtubules in subsequent meiosis I. The bouquet-deficient strains (e.g., *bqt1*Δ) tend to lose CENP-A (the centromere-specific variant of histone H3) and Swi6 [the heterochromatin protein 1 (HP1) ortholog] at centromeres, indicating that the telomere bouquet is required for the maintenance of heterochromatin during meiosis (Klutstein et al., 2015).

It is also reported that telomere bouquet is required to activate the cyclin-dependent kinase-cyclin B (CDK-cyclin B) at SPBs at the later stage of meiotic prophase (Moiseeva et al., 2017). Bouquet-deficient cells also show defects in the detachment of centromeres from SPBs (illustrated in Figure 1D), indicating that the detachment of centromeres and the collection of telomeres toward SPBs are linked to each other by the LINC complex and microtubules (Katsumata et al., 2016).

Further investigation to address the biological meaning of telomere bouquet, except for pairing of homologous chromosomes, will clarify how the conserved chromosomes’ behavior functions for the production of gametes.

## Composition of Kinetochores and Spindle Poles Is Altered in Meiosis

Another essential phenomenon seen during the initial stage of meiotic events is reorganization of kinetochore and SPB components.

In fission yeast, most of the kinetochore components, including both inner and outer factors, stably constitute kinetochores throughout the mitotic cell cycle. There are only few exceptions: the Dam1 complex is a mitosis-specific kinetochore component, while the Mis18 complex disappears in mitosis (Hayashi et al., 2004, 2014; Liu et al., 2005; Hirai et al., 2014; Subramanian et al., 2014). Components of fission yeast tend to be constitutive in contrast to those of metazoans. The modification of kinetochore proteins by mitotic kinases might have been developed during the evolution from yeast to metazoans. In meiotic prophase, however, most of the outer kinetochore components dismiss, including the Ndc80 (also known as Hec1) complex, the Mis12/MIND complex and Spc7 (KNL1), whereas inner factors remain intact (“slim” kinetochores; Figure 1D; Goshima et al., 1999; Wigge and Kilmartin, 2001; Kerres et al., 2004; Asakawa et al., 2005; Hayashi et al., 2006; Meyer et al., 2015).

Signaling *via* the mating pheromone–MAPK pathway in the early stage of meiosis is responsible for the disappearance of the kinetochore components. A reason for making slim kinetochores is for the detachment of centromeres from SPBs (Figure 1D), through which chromosomes get inverted to promote pairing and recombination.

In budding yeast meiosis, kinetochores detach from the SPB as in fission yeast meiosis. Although kinetochore detachment in fission yeast is mediated by the delocalization of outer kinetochore complexes, that in budding yeast is controlled through the degradation of Ndc80/Hec1 (Chen et al., 2020). It is suggested that the dissociation of outer kinetochore components may trigger the recruitment of Mam1 (monopolin) to kinetochores for mono-orientation at meiosis I (Meyer et al., 2015).

It is intriguing to point out that many of SPB components including Polo kinase alter its localization during meiotic prophase as is observed for kinetochores (“slim SPB” in Figure 1D; Ohta et al., 2012). SPB components are categorized into three groups: (i) disappeared from SPBs during prophase: Cut12, Pcp1, and Spo15. Although Plo1 is not a constitutive component of SPBs (Ohkura et al., 1995; Mulvihill et al., 1999; Tanaka et al., 2001), Plo1 predominantly localizes to SPBs during mitosis, whereas not during meiotic prophase. Plo1 localizes instead to kinetochores as mentioned above. It gets localized to SPBs later, upon entry into meiosis I. Therefore, Plo1 can be categorized to (i); (ii) reduced amount of proteins at SPBs: Sid4 and Cdc11 (Tomlin et al., 2002; Morrell et al., 2004); and (iii) constantly localized to SPBs: Cdc31, Sfi1, Sad1, and Ppc89—those mainly function for SPB duplication as SPB half-bridge factors (Hagan and Yanagida, 1995; Kilmartin, 2003; Paoletti et al., 2003; Rosenberg et al., 2006; Bouhlel et al., 2015).

The slimming down of SPB components shares similarities with that of kinetochore components. For example, the timing of dis/re-appearance of both factors is synchronized, and particularly the disappearance of both factors is dependent on the mating pheromone–MAPK pathway. One of the physiological meanings of SPB reorganization is to avoid overduplication of meiotic SPBs by temporarily reducing Plo1 from SPBs, as enforced localization of Plo1 to meiotic SPBs results in an excess of the SPB number (Ohta et al., 2012; Agarwal et al., 2018). Slim SPB might uncouple SPB duplication with DNA replication in meiosis, which potentially explains how cells secure two rounds of SPB duplication with oscillation of CDK activity in meiosis. Another advantage for dynamic SPB reorganization is to temporarily deposit Plo1 to kinetochores at meiosis I onset: reducing Plo1 at SPBs to get priority to depositing it at kinetochores. The kinetochore localization of Plo1 plays crucial roles in the collection of dispersed kinetochores before meiosis I entry (see below).

The slimming down of SPBs is not evident in budding yeast meiosis, although only a small number of SPB-associated proteins fluctuate: for instance, a meiosis-specific *S. cerevisiae* protein Ndj1 dissociates from SPBs in meiotic prophase (Li et al., 2015). The discrepancy in yeast species might be due to SPB structures. A budding yeast SPB is a three-layered structure and is embedded to the nuclear envelope throughout the cell cycle, whereas a fission yeast SPB has an amorphous structure and is inserted into the nuclear envelope prior to M phase entry (Ding et al., 1997; Jaspersen and Winey, 2004). It is possible that fission yeast SPB is structurally flexible compared to that of budding yeast and that the plasticity may allow a reorganization of the components.

Thus, in mitosis, the components of kinetochores and SPBs are almost constitutive, whereas there are a number of reorganizations taking place probably to streamline cellular machineries to adapt for divisions specialized for the production of gametes. Data remain elusive regarding molecular mechanisms as to how slimmed kinetochores and SPBs are rebuilt right before entry into meiosis I. Previous studies indicated the requirement of activities of cell cycle kinases. Re-accumulation of SPB components including Plo1 at meiosis I onset requires elevation of the CDK activity (Ohta et al., 2012). In budding yeast, the activity of the Aurora B kinase Ipl1 is required for the reappearance of Ndc80 to kinetochores before meiosis I (Kim et al., 2013; Meyer et al., 2015). It is possible that those kinases phosphorylate some key components that are required for further recruitment of other components to reconstitute SPBs and kinetochores before entry into meiosis I.

## Entry Into Meiosis I: Position of Chromosomes Is Altered by Meiotic Microtubules

**FIGURE 2:**
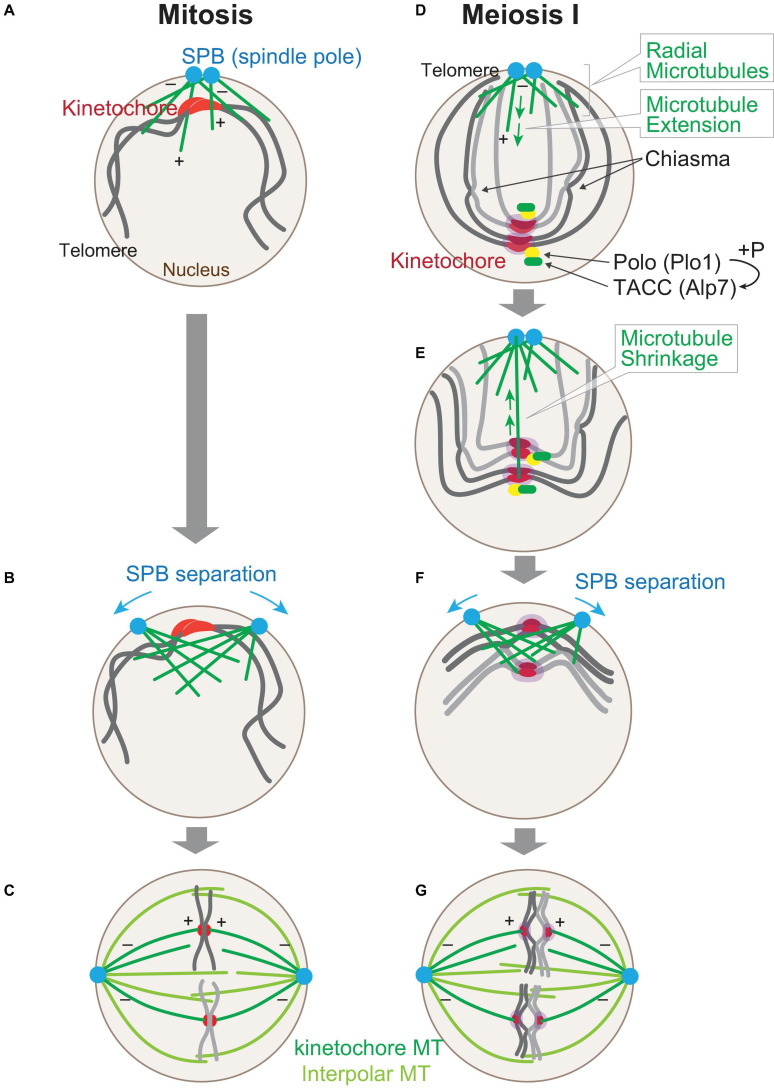
Chromosome arrangement changes upon sexual differentiation. Chromosome positioning upon entry into mitosis. At the onset of mitosis **(A)**, kinetochores (red) are clustered at spindle pole bodies (SPBs; blue), where microtubules (green) start to nucleate. The polarity of a microtubule is indicated (+, –). SPBs start to separate as the microtubules grow **(B)**, and in metaphase **(C)**, kinetochore microtubules (MTs) and interpolar MTs comprise the nuclear spindle. **(D–G)** Chromosomes are re-arranged upon entry into meiosis I right after the horse-tail nuclear movement is ceased **(D)**. Microtubules are nucleated from SPBs, but kinetochores are scattered in the nucleus. Kinetochores are mono-oriented by Moa1 (Meikin, purple), which recruits Polo kinase (Plo1). Plo1 then recruits Alp7 (TACC) to pre-attached kinetochores. A radial array of microtubules is formed from SPBs **(E)** and capture kinetochores and retrieve them toward SPBs. Telomeres are dissociated from SPBs. At the timing when kinetochore retrieval is completed **(F)**, SPBs start to separate to assemble the bipolar spindle **(G)**. Note that homologous chromosomes are independently attached to microtubules in mitosis **(C)**, whereas they are paired in meiosis I **(G)**.

Next, we compare the chromosome arrangement in the nucleus upon entry into mitosis and into meiosis I. As discussed above, centromeres (kinetochores) are located close to SPBs in the interphase of the mitotic cell cycle (Figure 1C). This allocation is suitable for easy connection between microtubules and kinetochores upon entry into mitosis, as microtubules are nucleated from two SPBs, where kinetochores have been clustered even during interphase (Figure 2A).

The SPBs, duplicated in interphase, are separated from each other on the surface of the nucleus, as microtubules emanated from each of the SPBs start to overlap and interdigitate upon mitotic entry (Figure 2B). Finally, those two SPBs are separated to opposite sides so that the bipolar spindle can be assembled by metaphase (Figure 2C).

On the contrary, in cells entering meiosis I, positioning of chromosomes is completely upside-down as a result of centromere dissociation and telomere clustering. In addition, chromosomes are duplicated as sister chromatids, and homologous chromosomes are paired with chiasmata as a result of meiotic recombination during the horse-tail nuclear movement, as illustrated in Figure 2D. The upside-down positioning of chromosomes could be a potential risk for the subsequent chromosome segregation in meiosis I because kinetochores are located distal from the microtubule nucleation site, unlike that in mitosis.

When cells enter meiosis I, the horse-tail nuclear movement ceases, and microtubules are nucleated from SPBs toward inside the nucleus as observed at mitotic entry. In contrast to mitotic entry, meiotic cells, at this stage, start to nucleate an extensive radial array of microtubules from SPBs (Figure 2D; Kakui et al., 2013).

The extended microtubules then capture kinetochores scattered in the nucleus and then shrink to retrieve the attached kinetochores toward SPBs (Figure 2E). The retrieval of kinetochores mostly relies on depolymerization of microtubules rather than sliding of kinetochores on microtubules. First, a kinetochore may attach to the lateral surface of a microtubule, and this can then be converted to end-on pulling when the plus end of the shrinking microtubule reaches the kinetochore. End-on pulling motion in meiotic kinetochore retrieval relies on the Dam1 complex, which forms oligomeric rings around microtubules (Westermann et al., 2005). In contrast, budding yeast kinetochores are mainly collected by sliding on the surface of microtubules in mitosis (Tanaka et al., 2005b). Homologous kinetochores are retrieved as a pair by microtubules, and mathematical modeling indicated that the dynamic instability of microtubules is essential for efficient retrieval and that paired configuration of kinetochores accelerates the capture by pivoting microtubules (Cojoc et al., 2016; Blackwell et al., 2017).

When homologous kinetochores are captured and pulled by microtubules, the mode of attachment may be mostly monopolar, the state in which both kinetochores are pulled by microtubules emanated from the same spindle pole. This may be resolved by Aurora B kinase, as budding yeast meiotic kinetochores retrieved in a monopolar manner are converted to bipolar attachment through phosphorylation by the Aurora B kinase Ipl1 (Meyer et al., 2013). The kinetochore protein Dam1 is also shown to promote chromosome bi-orientation through phosphorylation by the Mps1 kinase (Meyer et al., 2018).

During retrieval of kinetochores by pivoting monopolar microtubules, formation of the bipolar spindle (separation of two SPBs) should be repressed, although the system that surveys the completion of the scattered kinetochores does not appear very strict, as occasionally bipolar spindle starts to assemble even before the completion of kinetochore retrieval (Kakui et al., 2013). At least in budding yeast meiosis, the Aurora B Ipl1 localizes to SPBs during meiotic prophase and is involved in delaying the assembly of bipolar formation driven by S-CDK (Kim et al., 2013; Newnham et al., 2013).

Regarding regulation of SPB separation, a meiosis-specific telomere-associated protein, Ndj1, is known to localize to SPBs together with Mps3 (a SUN-domain protein) in budding yeast meiosis to suppress the premature separation of SPBs (Li et al., 2015, 2017). Polo kinase Cdc5 removes Ndj1 from the half-bridge structure connecting two SPBs, thereby promoting SPB separation in meiosis I. As Ndj1, Mps3, and Csm4 are also involved in telomere positioning and motility in meiotic prophase, loss of Ndj1 in meiosis I brings two consequences: SPB separation and telomere dissociation from the nuclear envelope (Conrad et al., 2008; Kosaka et al., 2008; Wanat et al., 2008; Li et al., 2015), indicating that these factors may play central roles to coordinate mitotic progression and chromosome configuration.

In summary, the extensive microtubules are assembled to relocate chromosomes to the original position as seen in mitotic entry, thereby minimizing the potential risk of segregation errors in meiosis I.

In addition to the assembly of radial microtubules, cells at meiosis I onset take the second strategy, namely, cells utilize Alp7 (also known as Mia1), the *S. pombe* ortholog of the microtubule-associated protein transforming acidic coiled-coil protein (TACC) for this purpose. Alp7 primarily localizes to SPBs and microtubules. Alp7 also localizes to mitotic kinetochores once captured by spindle microtubules, which means that Alp7 is delivered to kinetochores by microtubules and stabilizes kinetochore–microtubule attachment in mitosis (Oliferenko and Balasubramanian, 2002; Sato et al., 2003, 2004; Sato and Toda, 2007). Although Alp7 localizes also to meiotic kinetochores, it is of note that Alp7 localizes there even before microtubule attachment (Figure 2D). Alp7 precociously localized to scattered kinetochores promotes capture by radial microtubules (Kakui et al., 2013).

Thus, cells employ two machineries—extension of radial microtubules and precious localization of Alp7 to kinetochores— to synergistically promote relocation of chromosomes. Do these machineries operate also during mitosis or only during meiosis? The radial array of microtubules is not evident in cells at mitotic onset, in which kinetochores are constantly located in the vicinity of SPBs. When kinetochores are artificially detached from SPBs upon entry into mitosis, for example, by the use of transient exposure to microtubule poisons, similar long microtubules are assembled after drug washout to capture and collect the scattered kinetochores. Thus, the machinery utilizing extending microtubules may also operate during mitosis as a backup system to respond to the unexpected risk, although it has not been clarified if the molecular mechanisms for microtubule extension are identical in mitosis and in meiosis. Alternatively, either SPB separation or maturation in meiosis I could be repressed by slim SPBs during meiotic prophase to efficiently form a radial microtubule array.

In meiosis, however, extension of microtubules is observed in cells at the stage without exception, and the microtubules complete kinetochore retrieval mostly by the time SPBs start to separate (Figures 2F,G), suggesting that the scheme in meiosis is programmed as a physiological system rather than as a reflex action to the contingency. The second strategy, namely, the precocious deposition of Alp7 to microtubule-free kinetochores, is exclusively observed in this stage, and a similar localization cannot be observed in mitotic cells. Thus, deposition of Alp7 to pre-attached kinetochores is programmed specifically for meiosis. This is indeed evidenced by the molecular mechanism underlying the precocious localization of Alp7 to meiotic kinetochores: the meiosis-specific localization of Alp7 is dependent on the Polo kinase Plo1, which is also located to pre-attached kinetochores in meiosis (Figure 2D).

As mentioned above, Plo1 localizes to pre-attached kinetochores using Moa1 (Meikin) as a platform; therefore, Alp7 localization to the kinetochores is also a meiosis-specific event. Taken together, we consider that Moa1–Plo1 plays the third function in meiosis—at the onset of meiosis I, kinetochores are highlighted as center for microtubule control: Moa1 (Meikin) recruits Plo1 (Polo kinase), which deposits Alp7 (TACC) to stably capture microtubules emanated radially from spindle poles.

Moa1–Plo1 has an additional role: Plo1 at meiotic kinetochores also phosphorylates Spc7 (KNL1) of the outer kinetochore components. This affects the localization of Bub1 kinase which is known as a checkpoint kinase and phosphorylates histone H2A to recruit shugoshin at centromeres (Tang et al., 2004; Kawashima et al., 2010). In mitosis, the kinetochore localization of Bub1 is transient, whereas Bub1 in meiosis persists at kinetochores until anaphase of meiosis I because Spc7, the platform for Bub1, is phosphorylated by Plo1 specifically in meiosis (Miyazaki et al., 2017).

Thus, Moa1–Plo1 plays a central role to dictate a number of meiosis-specific events regarding the interaction of kinetochores and microtubules, thereby differentiating meiosis from mitosis.

The progression of kinetochore–microtubule association is monitored by the spindle assembly checkpoint (SAC) machinery in mitosis and meiosis. Briefly, kinetochores unattached to microtubules are recognized by the Mad1–Mad2 complex, the main components of SAC. When chromosomes are repositioned at the onset of meiosis I, the unattached kinetochores are not recognized by Mad1–Mad2. This is probably due to a lack of sufficient CDK activity, which is a prerequisite for the localization of Mad1–Mad2 to unattached kinetochores (Aoi et al., 2014).

For an entire resolution of the bouquet arrangement, telomeres that have been clustered around SPBs during meiotic prophase are detached from SPBs upon entry (Figure 2E), although the molecular mechanism remains elusive. Resolution of telomere clustering occurs almost at the same timing with kinetochore retrieval, albeit slightly later than the retrieval. The resolution requires elevation of the cyclin-dependent kinase activity by Cdc25 phosphatase, which is transcriptionally activated by the meiosis-specific transcription factor Mei4 (Murakami-Tonami et al., 2007; Kakui et al., 2011, 2013). Cdc13 (cyclin B) predominantly accumulates at bouquet telomeres for the resolution of telomere clustering (Moiseeva et al., 2017).

## Power Balance for Spindle Pole Separation in Mitosis and Meiosis

It has been recently shown that difference in chromosome configuration in mitosis and meiosis affects bipolar spindle organization using their kinetic properties.

The assembly of bipolar spindle is based on the elongation of microtubules and their mutual and physical interaction. Spindle microtubules are emanated from both of the two SPBs, and they interact with each other to separate the SPBs outward, which is the major driving force for bipolar spindle formation.

**FIGURE 3:**
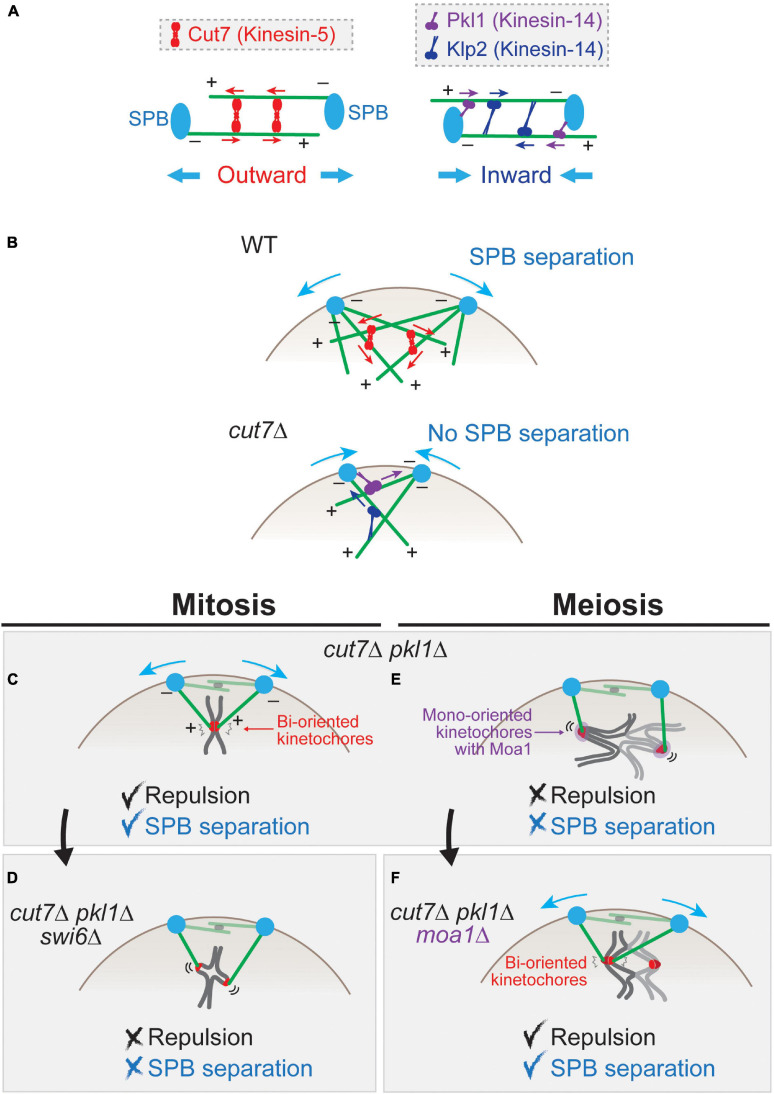
Force balance affects spindle pole body (SPB) separation in mitosis and meiosis. **(A)** Schematics for functions of Cut7 (kinesin-5) and Pkl1 and Klp2 (kinesin-14) for the inter-SPB distance. A tetramer of Cut7 (red) captures two bundles of microtubules. When they are aligned in an anti-parallel manner, the plus-end-directed Cut7 generates the outward force that consequently separates two SPBs. The polarity of microtubules (+, –) is indicated. Microtubules are tethered to SPBs at their minus ends. Pkl1 localizes to SPBs and Klp2 to the microtubules. Those minus-end-directed motors generate the inward force. **(B)** SPB separation in prometaphase of mitosis. In wild-type cells, the microtubules nucleated from two SPBs are linked by Cut7 and separate the SPBs. In the *cut7* deletion (*cut7*Δ) mutant, SPBs fail to separate because of the inward force generated by Pkl1 and Klp2. **(C)** In mitotic *cut7*Δ *pkl1*Δ cell, SPBs are separated by repulsive forces generated by sister kinetochores. **(D)** When Swi6 (HP1) is deleted, the structure of sister kinetochores is loosened, which does not generate a sufficient repulsive force to separate SPBs. **(E)** In contrast to mitosis **(C)**, *cut7*Δ *pkl1*Δ cells in meiosis I cannot generate a sufficient repulsive force to separate SPBs. **(F)** When kinetochores are artificially bi-oriented by depleting Moa1 (*cut7*Δ *pkl1*Δ *moa1*Δ), sister kinetochores generate a repulsive force that causes SPB separation.

As illustrated in Figure 3A, a couple of kinesin motor proteins are involved in the separation of two SPBs. Kinesin-5 is a conserved subfamily of the kinesin superfamily motor proteins that move to plus-ends and functions as a homo-tetramer (Hagan and Yanagida, 1990, 1992; Kapoor et al., 2000). Cut7, the fission yeast ortholog of kinesin-5 subfamily members, is an essential protein required for outward SPB separation that functions as a tetramer (Hagan and Yanagida, 1990, 1992; Akera et al., 2015). Cut7 captures the lateral surface of a pair of interpolar microtubules emanating from both SPBs, and it moves toward their plus-ends along the microtubules. The motion consequently pushes two SPBs outward (Figures 3A,B).

On the contrary, members belonging to another subfamily kinesin-14 (Pkl1 and Klp2) are minus-end-directed and generate inward forces for SPBs (Figure 3A). Pkl1 preferentially localizes to SPBs and the spindle as well as the nucleoplasm during mitosis, and Klp2 localizes to spindle microtubules (Pidoux et al., 1996; Troxell et al., 2001; Simeonov et al., 2009).

The knock-out of Cut7 (*cut7*Δ) is lethal with an adjacent pair of SPBs, which extend the monopolar spindle therefrom. The lethality of *cut7*Δ and *cut7*-ts (temperature sensitive) mutants is suppressed by a simultaneous knock-out of pkl1 (Rodriguez et al., 2008; Syrovatkina et al., 2013; Olmsted et al., 2014; Syrovatkina and Tran, 2015). This can be explained in such a way that, in the absence of Cut7, the major force operating between two SPBs is inward force generated by Pkl1 and Klp2, which hampers SPB separation (Figure 3B). In the absence of two antagonistic kinesins Cut7 and Pkl1, the outward force wins again to consequently separate the SPBs (mitosis, Figure 3C).

This also indicates that there are additional factors that generate the outward force to separate SPBs other than Cut7. One of such factors is the microtubule-associated protein Ase1 (human PRC1), which is known to connect a pair of interdigitating microtubules as an anti-parallel bundle (Pellman et al., 1995; Loïodice et al., 2005; Yamashita et al., 2005). This indicates that Ase1 connects interpolar microtubules in *cut7*Δ *pkl1*Δ mitosis (Figure 3C), and microtubule polymerization by Alp14 (a member of the ch-TOG/XMAP215/Dis1 microtubule-associated protein family) together with Alp7 (TACC) pushes the SPB of the other side outward (Yukawa et al., 2017). Similarly, other microtubule-associated proteins promote outward force generation in the absence of Cut7 (Yukawa et al., 2019).

In addition to microtubule-associated proteins, chromosome is another factor that generates outward force for SPB separation. In *cut7*Δ *pkl1*Δ mitosis (Figure 3C), a pair of sister chromatids mediate pole-to-pole connection through kinetochore–microtubule attachment (Shirasugi and Sato, 2019). The microtubules use the sister kinetochores as the fulcrum to generate the repulsive force which separates SPBs.

This is evidenced by genetic analyses; for instance, SPB separation is inhibited when the mitotic cohesin Rad21 is removed (i.e., in the *cut7*Δ *pkl1*Δ *rad21-ts* triple mutant). Moreover, when the sister kinetochores are unfastened by reduction of centromeric cohesion using deletion of Swi6 (HP1) (Ekwall et al., 1995; Bernard et al., 2001; Nonaka et al., 2002; Kitajima et al., 2003), the outward force is significantly diminished (Figure 3D; Shirasugi and Sato, 2019). These results altogether demonstrate that centromeric cohesion and functional sister kinetochores are required for generation of the outward force in the absence of Cut7 and Pkl1.

In contrast to mitosis, *cut7*Δ *pkl1*Δ cells are not able to separate SPBs in meiosis I (Shirasugi and Sato, 2019). This is due to the loosened connection between homologous kinetochores instead of a tight sister kinetochore connection of mitotic cells (Figure 3E). When mono-orientation of sister chromatids is converted to bi-orientation by deletion of Moa1 (i.e., *cut7*Δ *pkl1*Δ *moa1*Δ cells), SPBs are separated (Figure 3F).

This provides us two concepts. First, the rigidity of the kinetochore connection matters because it determines whether an additional outward force for SPB separation is generated in mitosis and in meiosis. Second, the kinetochore-mediated outward force is weaker in meiosis I than in mitosis, owing to meiotic kinetochore mono-orientation. This may lead to a delay in SPB separation in meiosis I, unless the Cut7-mediated force is somehow augmented or the opposing inward force by kinesin-14 motors decreases. Alternatively, SPB separation may be suspended until scattered kinetochores are retrieved near SPBs. When kinetochores are retrieved to the close vicinity of SPBs, it may be able to generate a rigid repulsive force by short microtubules that is sufficient for SPB separation. This may be reasonable for cells at this stage, as they need to earn some additional time until all the scattered kinetochores are collected to SPBs. Thus, the kinetochore-mediated repulsive force may modulate the balance of mechanical forces, through which meiosis-specific cell cycle progression and chromosomal events may be timely coordinated.

In general, either in mitosis or meiosis, fission yeast microtubules do not complete end-on attachment to kinetochores by the timing of SPB separation. Therefore, the kinetochore-mediated SPB separation may not rely on the end-on attachment; rather, a pair of bi-oriented kinetochores serves as a glue factor that connects two anti-parallel microtubules through attachment to their lateral surfaces, similarly to the microtubule-associated bundling factor Ase1.

Kinetochore-driven centrosome separation has also been observed in HeLa cells. When a kinetochore protein, either CENP-O (Mcm21) or CENP-L, is depleted, separation of centrosomes is delayed albeit partially, and this is due to defects in the formation of kinetochore microtubules (kinetochore fibers or k-fibers) (Toso et al., 2009; McHedlishvili et al., 2012).

There are two major pathways for centrosome separation in HeLa mitosis: the aurora A-dependent pathway, which is presumably for centrosomal microtubule-mediated separation, and the kinetochore-dependent pathway (Toso et al., 2009). The kinetochore-mediated pathway does not exert a significant influence on mitotic progression when centrosomes have already been separated before nuclear envelope breakdown (∼50% of total mitotic cells), suggesting that the kinetochore-driven machinery is a backup for efficient centrosome separation in HeLa mitosis.

When the nuclear envelope breakdown precedes centrosome separation in prometaphase, lateral attachment and kinetochores to microtubules and their lateral transport are promoted to form a ring-like alignment of chromosomes, called prometaphase rosette (Nagele et al., 1995; Bolzer et al., 2005). The prometaphase rosette is gradually converted to metaphase congression through the transport of laterally attached kinetochores by the kinesin-7 motor CENP-E and the chromokinesin Kid (Tokai et al., 1996; Funabiki and Murray, 2000). Kinetochore-driven centrosome separation may occur during the conversion and establishment of the metaphase alignment. These observations imply that the way of kinetochore-mediated SPB separation is an analogous phenomenon to the similar centrosome separation.

During acentrosomal meiosis I of mouse oocytes, the Ndc80 complex of outer kinetochores accumulate the microtubule crosslinker Prc1 (yeast Ase1) to kinetochores, which becomes a center for microtubule bundling to assemble the functional bipolar spindle even without positional cues at spindle poles (Yoshida et al., 2020). Thus, the kinetochore-mediated force generation, as well as the Ase1/Prc1-dependent cross-linking pathway in yeast mitosis, may be an evolutionary origin for spindle organization in female acentrosomal meiosis. In line with this, it is recently found that acentrosomal spindle microtubules containing Ase1/Prc1 can be induced in fission yeast meiosis (Pineda-Santaella and Fernández-Álvarez, 2019).

## Anaphase Events: Spindle Elongation and Resolution of Recombination

As mentioned above, the SAC machinery monitors the state of kinetochore–microtubule interaction, and in the case of problems, SAC arrests cell cycle progression in metaphase. SAC detects two types of erroneous interactions: an improper attachment and a lack of tension between kinetochores (Nezi and Musacchio, 2009). The overall functions of SAC are common in mitosis and in meiosis, but tension is generated in a different manner. In mitosis, tension by microtubules is generated between sister kinetochores (left, Figure 1B), whereas it is generated between homologous kinetochores (right).

In anaphase I, homologous chromosomes with chiasmata are segregated; hence, chiasmata need to be resolved by anaphase onset. The resolution of meiotic recombination intermediates is promoted by the Skp1–Cul1-F–box (SCF) complex. SCF constitutes a conserved ubiquitin ligase family and contributes a number of cellular phenomena, and the fission yeast orthologs of the components are Skp1, Cul1, and at least 18 F-box proteins [reviewed in Toda et al. (1999)]. In the temperature-sensitive mutant of SCF–Skp1 (*skp1*-ts), the anaphase spindle becomes abnormally bent in the nucleus, both in mitotic and meiotic anaphase (Lehmann and Toda, 2004; Okamoto et al., 2012).

The bend spindle in anaphase I is due to unresolved meiotic recombination intermediates that remained until anaphase as evidenced by the prolonged foci of Rad51 (the RecA homolog) indicating sites of meiotic recombination (Muris et al., 1993; Shinohara et al., 1993). When meiotic cohesin Rec8 or the meiotic endonuclease Spo11 (the fission yeast ortholog is named Rec12) is deleted, the bent spindle phenotype is suppressed, verifying that entangled chromosomes by prolonged recombination intermediates attached to microtubules hamper the full elongation of the anaphase spindle; therefore, the spindle is bent.

In conclusion, Skp1 and the F-box helicase Fbh1 are required for the resolution of meiotic recombination intermediates, although it remains to be elucidated which protein is targeted by SCF-Skp1–Fbh1 for degradation for the resolution (Okamoto et al., 2012; Tsutsui et al., 2014).

**FIGURE 4:**
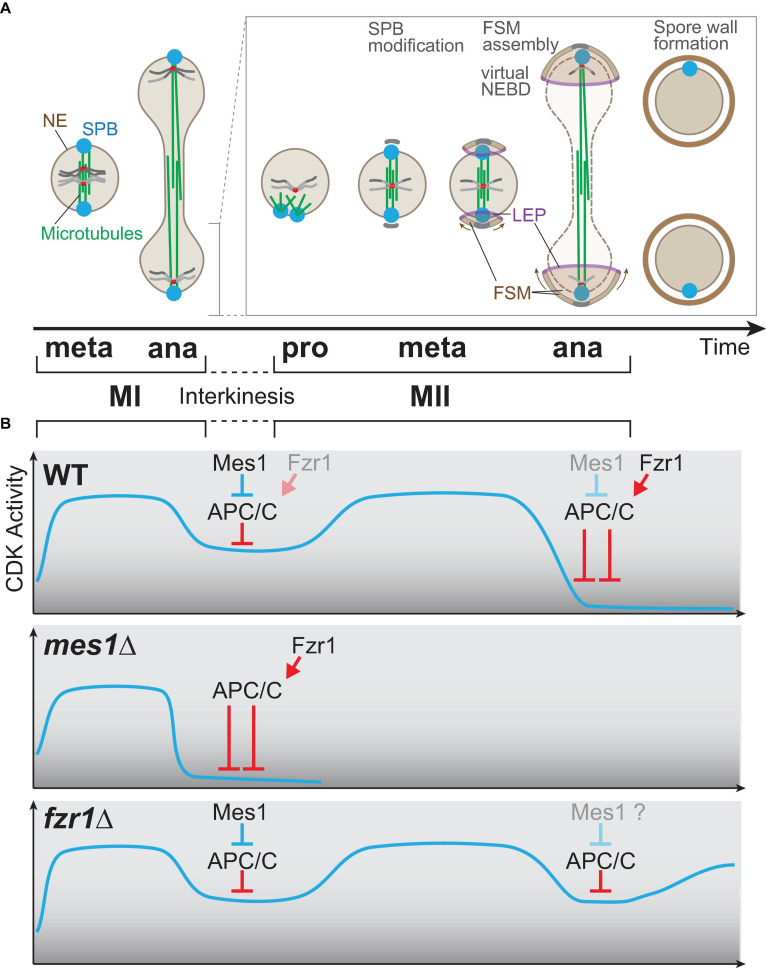
Meiosis-specific cell cycle progression from meiosis I to meiosis II. **(A)** A graphical view of meiotic progression from metaphase of meiosis I (MI) to anaphase of meiosis II (MII). After anaphase I, only one of two nuclei is chosen for drawing to illustrate MII progression. In prophase II, the microtubules are nucleated from spindle pole bodies (SPBs) and form the bipolar spindle for meiosis II as in mitosis and MI. At the transition stage of metaphase to anaphase, each SPB is modified, and the globular forespore membrane (FSM) begins to grow to surround the nucleus. The leading edge of the FSM opening is decorated by leading edge proteins. During anaphase II, the barrier function of the nuclear envelope is invalidated, which is an incident called virtual nuclear envelope breakdown. After completion of MII, the rigid spore wall is assembled. **(B)** The kinetics of the CDK activity during meiosis. The horizontal axis (time) is shared with the time scale in **(A)**. In wild-type cells (top), the CDK activity elevates until metaphase I and drops at anaphase I onset, which is triggered by APC/C. The APC/C inhibitor Mes1 modulates the activity of APC/C to a moderate level so that cells can enter anaphase I and to restart meiosis II, which requires re-accumulation of the CDK activity. In *mes1*Δ cells (middle), Fzr1, an APC/C coactivator, is prematurely activated to fully activate APC/C, and the cells cannot enter meiosis II, and terminates meiosis early instead. In *fzr1*Δ cells (bottom), meiosis I proceeds almost normally, but CDK repression after anaphase II onset is not sufficient as APC/C cannot be fully activated in the absence of Fzr1. The cells then start the aberrant third division albeit incomplete.

The function of SCF–Skp1 in the resolution process appears conserved in eukaryotes: the *Arabidopsis ask1* mutant (Ask1 is the Skp1 ortholog) has the spindle which displays an overall normal structure but somewhat longer than that of WT cells during meiosis I (Yang et al., 1999; Yang and Ma, 2001; Wang et al., 2004). The difference in spindle morphology (bent or long) in these two organisms could be simply due to whether the spindle poles are embedded in the nuclear envelope and whether the spindle is assembled in the compartmentalized nucleus in closed mitosis (Figure 4A), and the function in resolution of meiotic intermediates is likely to be conserved.

## Exit From Meiosis I and Entry Into Meiosis II

One of the most enigmatic mechanisms of meiosis is two consecutive rounds of cell division: meiosis I (MI) and meiosis II (MII) without replicating DNAs, which is a clear contrast to the single M phase per cell cycle in mitotically growing cells. The interval between MI and MII is called the interkinesis period.

After segregation of homologous chromosomes and spindle elongation in anaphase I of fission yeast (Figure 4A), the spindle is disorganized once, and after a while (∼30 min at 25°C), the spindle for meiosis II starts to assemble (prophase of MII, Figure 4A; Sato et al., 2009). Specialized regulation of CDK is essential for the interkinesis period, followed by the initiation of meiosis II. Earlier genetic studies have demonstrated that the CDK activity is essential to start meiosis II, as the *cdc2/tws1* mutant cannot enter meiosis II, and terminates meiosis with the formation of the spore wall (Nakaseko et al., 1984; Grallert and Sipiczki, 1990; Iino et al., 1995).

The drug-sensitive mutant *cdc2-as* (analog-sensitive) (Dischinger et al., 2008) contains a mutation in the gatekeeper residue, which causes a reduction of the Cdc2 activity. The *cdc2-as* mutant is deficient in meiosis II initiation and terminates meiosis in a binucleate state even without exposure to the ATP analog. The activity of the mutant protein can be regained by the introduction of a suppressor mutation into the Cdc2-as protein. The *cdc2-asM17* mutant has additional mutations to improve the Cdc2-as activity and proceeds meiosis normally to produce normal spores (Aoi et al., 2014).

The *mes1* mutant has been isolated as a *mes* (meiosis second) mutant defective in meiosis II, although the *mes1* mutant does not display the spore wall unlike *cdc2/tws1* (Bresch et al., 1968; Shimoda et al., 1985). The *mes1* gene is expressed specifically during meiosis and encodes an inhibitor of the anaphase promoting complex/cyclosome (APC/C) (Kishida et al., 1994; Izawa et al., 2005; Kimata et al., 2008), the mega-complex serving as a conserved E3 ubiquitin ligase [reviewed in Yamano (2019)]. In WT cells entering anaphase I, the CDK activity is maintained by Mes1, which blocks the full activation of APC/C to a moderate level that is sufficient to initiate anaphase I (top, Figure 4B).

The APC/C is, in general, activated by the coactivator Cdc20 (Slp1 in fission yeast) both in mitosis and meiosis, but in meiosis, Fzr1 (also known as Mfr1 and Sms1) also assists the activation of APC/C (Asakawa et al., 2001; Blanco et al., 2001; Kimata et al., 2011; Aoi et al., 2013). In WT meiocytes, Slp1 is the main coactivator for anaphase I onset, whereas Fzr1 is mainly for anaphase II onset and completion of meiosis. Mes1 binds and initially inhibits Fzr1 and Slp1 as a competitive substrate to prevent premature APC/C activation until anaphase I. Mes1 is a competitive substrate but not a pseudosubstrate for Slp1; therefore, Slp1 eventually overcomes the inhibition by Mes1 and triggers anaphase I onset (Kimata et al., 2008).

On the contrary, Mes1 serves as a pseudosubstrate for Fzr1; therefore, Fzr1 remains inactive possibly until Mes1 is somehow diminished. In the absence of Mes1 (*mes1*Δ), APC/C is prematurely activated by Fzr1 in anaphase I, which terminates meiosis early, right after anaphase I, without initiating meiosis II (middle, Figure 4B). In line with this, the early termination of *mes1*Δ can be substantially suppressed by the simultaneous deletion of Fzr1: the *mes1*Δ *fzr1*Δ double mutant initiates meiosis II and produces spores (Aoi et al., 2013).

The *fzr1*Δ single mutant can initiate anaphase I and anaphase II, as Slp1 functions as the major APC/C coactivator for both divisions, but for complete exit from meiosis II, Fzr1 is essential in addition to Slp1, as *fzr1*Δ meiocytes do not exit from meiosis even after anaphase II; cyclin B reaccumulates later instead (bottom, Figure 4B). The *fzr1*Δ mutant thus initiates “meiosis III”, although the division is incomplete in terms of insufficient materials such as SPBs and chromosomes.

The checkpoint (SAC) functions twice, namely, at meiosis I and II. The activity of SAC during two sequential divisions may be regulated in a continuous manner. When the first division is delayed by SAC, the anaphase onset of meiosis II is advanced, which means that the SAC effect was reduced at meiosis II to compensate the previous delay that occurred in meiosis I (Yamamoto et al., 2008).

In conclusion, the number of meiotic divisions is exclusively determined as two, neither one nor three. This biological rule strictly conserved and complied among eukaryotes is defined by the repetitive battles of the “CDK–APC/C derby”, which is reinforced by meiosis-specific factors Mes1 and Fzr1.

Although the seesaw battle is commonly seen in meiocytes of any species, the underlying molecular mechanisms may be divergent. The functional homologs of *S. pombe* Mes1 are OSD1 in plants and Erp1/Emi2 in vertebrates (Liu et al., 2006; Ohe et al., 2007; d’Erfurth et al., 2010; Cromer et al., 2012). In oocyte meiosis of vertebrates, Erp1 functions as a cytostatic factor that arrests the meiotic cell cycle in metaphase II (Masui and Markert, 1971; Inoue et al., 2007; Nishiyama et al., 2007), although fission yeast meiosis does not particularly arrest at metaphase II.

*Arabidopsis* OSD1 (*OMISSION OF SECOND DIVISION 1*) is also an APC/C inhibitor, which ensures initiation of meiosis II together with TAM (Cyclin A; *TARDY ASYNCHRONOUS MEIOSIS*), and the *tam osd1* double mutant cannot initiate meiosis II (d’Erfurth et al., 2010). *Arabidopsis* TDM1/MS5 (*THREE DIVISION MUTANT 1/MALE STERILE 5*) is the functional homolog of Fzr1 and is required for exit from meiosis. The *tdm1/ms5* mutant exhibits the aberrant third meiosis similar to the *S. pombe fzr1*Δ mutant (Ross et al., 1997; Glover et al., 1998). Although the players for the CDK–APC seesaw battle appears conserved in fission yeast and plant cells, the way of molecular regulation seems distinct. For meiotic exit, the active level of fission yeast Fzr1 may be regulated transcriptionally, but the plant TDM1 is post-translationally regulated through phosphorylation (Cifuentes et al., 2016).

The other *S. pombe mes* mutant *mes2* is allelic to the *spo5* mutant, and the *spo5* gene encodes a meiosis-specific RNA-binding protein (Bresch et al., 1968; Kasama et al., 2006; Okuzaki et al., 2010). Spo5 promotes progression of meiosis II through regulation of cyclin B (Arata et al., 2014; Togashi et al., 2014). At the moment, the *mes* mutants isolated to date are only two, and many things still remain to be elucidated: e.g., how the *mes1* expression is controlled. It is reported that the *mes1* gene is spliced only during meiosis (Kishida et al., 1994; Shimoseki and Shimoda, 2001; Malapeira et al., 2005). Although the mechanism remains unclear, this may be dependent on the meiosis-specific splicing machinery particularly driven by the promoter region and transcription factors belonging to the forkhead family (Averbeck et al., 2005; Moldón et al., 2008).

## Meiosis II Is Not Just an Analogous Event to Mitosis

### Forespore Membrane Formation

An *S. pombe* diploid meiocyte produces two nuclei after anaphase I; each of the nuclei is next divided into two in meiosis II to finally produce four haploid spores (Figure 4A). The second division of meiosis is similar to mitosis regarding the pattern of chromosome segregation (equational division), in which sister chromatids are segregated. Besides that, meiosis II is generally linked with gametogenesis, which corresponds to sporulation in yeast species.

The detailed processes of sporulation and underlying molecular mechanisms are comprehensively reviewed in Shimoda (2004); therefore, here we briefly summarize the general picture of sporulation events.

In pro-prometaphase of meiosis II, SPBs separate to form the bipolar spindle. At the same time, SPBs get modified by the SPB component Spo15 so that the forespore membrane can be assembled from the modified SPBs (Ikemoto et al., 2000; Figure 4A). The forespore membrane gradually grows to surround and cover the nucleus from both SPBs, and the edge of the opening region of the forespore membrane is entirely decorated by the leading edge proteins (LEPs) including Meu14 (Okuzaki et al., 2003; Figure 4A). Growth of the forespore membrane is guided by LEPs and septins over the anaphase nucleus, and the opening closes by contraction, thereby completely surrounding the divided nuclei (Onishi et al., 2010; Yang et al., 2020; Figure 4A). The hard spore wall is then formed after completion of the forespore membrane.

### Virtual NEBD in Meiosis II

Observations in the last decade revealed that the sporulation events give some unexpected impacts on the progression of meiosis II. Here we focus on an interesting behavior of the nuclear envelope: virtual nuclear envelope breakdown (vNEBD). Both *S. pombe* and the budding yeast *S. cerevisiae* undergo closed mitosis in which the nuclear envelope persists in mitosis, in contrast to open mitosis seen in higher eukaryotes (Boettcher and Barral, 2013; Dey et al., 2020), but in meiosis II, this “closed” rule seems to be obscure: the nuclear envelope in anaphase II shows both aspects of open and close mitoses; therefore, this phenomenon has been termed “virtual nuclear envelope breakdown (vNEBD)” (Asakawa et al., 2016). In anaphase II, nucleoplasmic proteins mostly dispersed, although observation of the nuclear envelope and the nuclear pore complex indicated that the nuclear envelope itself is not particularly disrupted or fragmented at least in fluorescence microscopy and in transmission electron microscopy (Arai et al., 2010; Asakawa et al., 2010).

One of the triggers of vNEBD in anaphase II may be related to the formation of the forespore membrane, which is also assembled at the same timing. The dispersal of nuclear proteins to the cytoplasm during anaphase II can be blocked in several *spo* gene mutants, which are involved in the assembly of the forespore membrane. When the nuclear envelope expands in anaphase II, the lipid components constituting the nuclear envelope may be in a shortage because the components may need to be preferentially used for the assembly of the forespore membrane. This idea is based on the fact that vNEBD does not occur when the vesicle transport pathway that conveys membrane components from Golgi to endoplasmic reticulum is inhibited by a drug (Arai et al., 2010). This implies the possibility that vNEBD may be caused by a shortage of nuclear envelope components, which results in an increase of membrane permeability only during anaphase II.

Interestingly, vNEBD accompanies nuclear entry of the cytoplasmic RanGAP protein (Asakawa et al., 2010, 2011). RanGAP (Rna1 in *S. pombe*) is expected to localize constantly to the cytoplasm to govern the Ran GTPase-dependent nucleocytoplasmic transport. The aberrant nuclear entry of RanGAP indicates that the nucleocytoplasmic transport is invalidated during meiosis II. Even when the nuclear envelope seemingly persists as in closed meiosis II, the nuclear conditions can be temporarily neutralized as is seen in open mitosis of higher eukaryotes.

The biological roles of vNEBD had been undefined, but recently it was shown to promote the maturation of spores through redistribution of the nuclear proteasome subunit Rpn11 to the cytoplasm (Yang et al., 2020). This means that vNEBD can be induced by sporulation events, which in turn feedbacks to promote spore maturation.

Further studies will illuminate the molecular mechanisms to trigger vNEBD as well as the biological significance of the phenomenon. As the interplay between the nuclear envelope and the genome contributes to the determination of cell fate [reviewed in Talamas and Capelson (2015)], it would be tempting to investigate the role of (v)NEBD for differentiation of cells in yeast and other species.

### Dispensable Interpolar Microtubules

Another unexpected aspect of sporulation events is the effect of the forespore membrane on the spindle of meiosis II.

In general, the spindle comprises three types of microtubules: kinetochore microtubules (kinetochore fibers, k-fibers) as mentioned earlier (see Figures 2C,G), interpolar microtubules connecting two spindle poles in an antiparallel manner, and astral microtubules extending outward of the spindle from the poles. In fission yeast, the majority of astral microtubules are formed in the cytoplasm from SPBs, and some bundles are in the nucleus (Zimmerman et al., 2004). Other two types are in the nucleus. Both kinetochore microtubules and interpolar microtubules are essential in mitosis and meiosis I, but interpolar microtubules are dispensable for the bi-directional segregation of chromosomes only in meiosis II (Akera et al., 2012). When interpolar microtubules are disrupted by microtubule poisons, the globular forespore membrane serves as an interpolar structure on their behalf to separate SPBs to assemble a bipolar apparatus and to separate two nuclei. The forespore membrane is guided by LEPs and septin proteins and grows from two SPBs, and two globular structures make a physical contact with each other in the middle of the nucleus in anaphase II, when the interpolar microtubules are destroyed by microtubule poisons. As a pair of the contacted forespore membrane grows, they gradually cleave and separate the anaphase nucleus into two, even though there is no spindle elongation in the conditions.

The forespore membrane-mediated nuclear division partly contributes to physiological meiosis II in the presence of normal microtubules, as SPB separation is perturbed in the *spo15* mutant lacking meiosis II-specific SPB modification as well as in the *meu14*Δ *spn6*Δ double mutant, showing the disorganized forespore membrane by depletion of both LEP and septin structures (Akera et al., 2012). Data remained elusive on whether kinetochore microtubules are also dispensable in meiosis II, although it is technically impossible at the moment to remove even the last traces of kinetochore microtubules, as some microtubules are resistant to canonical drugs [benzimidazole compounds such as MBC (carbendazim) and TBZ (thiabendazole)].

It is also reported that, in mitosis, microtubule-independent nuclear fission also occurs (Castagnetti et al., 2010). SPBs can separate in the absence of spindle microtubules when *cdc11* mutant cells (defective in cytokinesis) are exposed to microtubule poisons. It is also possible that a constant increase of the nuclear membrane components, which are supposed to be used for nuclear elongation in anaphase, caused an abundance in surplus in the absence of spindle elongation, resulting in aberrant nuclear fission (Castagnetti et al., 2010), given the case of vNEBD (Arai et al., 2010). Interestingly, nuclear fission requires F-actin. This is reminiscent of animal cells in which F-actin-dependent mechanisms promote spindle positioning and orientation [reviewed in Sandquist et al. (2011)].

The study strikingly showed that chromosome segregation is also fine to some degree. This might be also due to actin-dependent mechanisms as in bacterial cells in which chromosome segregation is driven by actin-like cytoskeleton. It is also possible that the segregation system utilizes any nucleoplasmic factors such as Csi1, as a material that connects mitotic SPBs and kinetochores even in the absence of microtubules, because Csi1 has been shown to connect SPBs and centromeres constantly in interphase (Hou et al., 2012).

It should be noted that no specific systems have been so far identified that ensure the equal segregation of sister chromatids in eukaryotes besides spindle microtubules. Currently, it is hard to completely rule out the possibility that very tiny residual microtubule seeds remain at SPBs even in the presence of the drug, as such tiny microtubule seeds might be able to connect SPBs and kinetochores clustered altogether at the mitotic onset. Once such attachments were made, kinetochore-mediated SPB separation might take place (similar to the situation in Figure 3C) to separate SPBs and segregate sister chromatids. It has been impossible to completely disrupt microtubules and inhibit regrowth by existing drugs; it would be intriguing to revisit these phenomena again when more effective drugs are invented in the future.

## Perspectives

The evolutionary origin of meiosis has been discussed from the viewpoint of the phenomena for a long period, and one of the most reasonable ideas must be that meiosis was evolved from mitosis (Simchen and Hugerat, 1993). Although meiosis is different from mitosis in many ways, one of the most essential characteristics in meiosis could be pairing of homologous chromosomes. Meiosis might have first evolved from mitosis through the acquisition of homolog pairing as an additional step (Wilkins and Holliday, 2009). As the molecular mechanisms have been illuminated in the last decades, the idea is getting realistic as evidenced by the genes involved in key events in meiosis. Most of the key events in meiosis appear to be conducted by meiosis-specific genes that are paralogous to those used in mitosis. Assuming that paralogous genes are generated *via* gene duplication in the long history of evolution, Spo11 (*S. pombe* Rec12) might have evolved from other topoisomerase genes as a copy specific for homolog pairing in meiosis, and this could be a key incident to acquire meiotic recombination and the following reductional division in meiosis I.

The meiotic cohesin Rec8 could likewise be originated from a duplicated copy of the mitotic cohesin Rad21. Fzr1, a meiosis-specific activator of APC/C, might have evolved from the mitotic one Slp1 (Cdc20). Those key factors might have defined the outline of meiosis as a newly acquired division system. In addition to those copied genes, meiosis-specific genes whose ancestors are currently unknown are also created to fine-tune meiotic events to the current state.

On the other hand, we also know that molecules or detailed molecular mechanisms in meiosis have been differentiated depending on species, although the whole system of meiosis *per se* is common among eukaryotes. The molecular mechanisms are thought to be fine-tuned in each organism depending on internal and external reasons such as the lifestyle and surrounding environment. Considering similarities and differences among species and in between two types of divisions, we will be able to converge the divergent mechanisms to explore the ultimate origin in the future.

## Author Contributions

MS conceived the framework of the entire manuscript. MS, YK, and MT wrote the manuscript. MS prepared the figures. All the authors contributed to the article and approved the submitted version.

## Conflict of Interest

The authors declare that the research was conducted in the absence of any commercial or financial relationships that could be construed as a potential conflict of interest.
